# Retraction Note: SULF2 overexpression positively regulates tumorigenicity of human prostate cancer cells

**DOI:** 10.1186/s13046-026-03833-0

**Published:** 2026-09-24

**Authors:** Carolina M. Vicente, Marcelo A. Lima, Helena B. Nader, Leny Toma

**Affiliations:** 1 Departamento de Bioquímica, Disciplina de Biologia Molecular, Universidade Federal de São Paulo, UNIFESP, Rua Três de Maio, 100 – 4° andar, Vila Clementino, São Paulo, SP CEP 04044-020 Brazil; 2 Department of Biochemistry, Institute of Integrative Biology, University of Liverpool, Liverpool, UK

**Retraction Note: J Exp Clin Cancer Res 34, 25 (2015)**

**https://doi.org/10.1186/s13046-015-0141-x**

The Editor-in-Chief has retracted this article. Concerns were raised regarding a number of figures, specifically:

- Figure 2A: two pairs of blots appear to overlap, MEDIUM Vector and SULF2, and CELL Vector and SULF2, when flipped horizontally;
- Figure 4C: CTRL NEG and SULF2 panels of DU-145 appear to overlap;
- Figure 1A: discrepancies between the original data and published image were noted by the Publisher.

The authors provided some source data on request from the Publisher, but this was insufficient to address the concerns raised. The Editor-in-Chief therefore no longer has confidence in the reliability of the data reported in the article.

Authors Carolina M Vicente and Leny Toma disagree to this retraction. Authors Marcelo A Lima and Helena B Nader have not responded to any correspondence regarding this retraction.

